# Supplementary material for: A physiologically-based digital twin for alcohol consumption—predicting real-life drinking responses and long-term plasma PEth
Source: NPJ Digit Med. 2024 May 3;7:112. doi: 10.1038/s41746-024-01089-6 (PMC11068902; doi:10.1038/s41746-024-01089-6)
Supplement: Supplementary file 2 — Reporting Summary [file 41746_2024_1089_MOESM2_ESM.pdf]

nature portfolio

Corresponding author(s): Henrik Podéus

Last updated by author(s): Mar 11, 2024

Reporting Summary

Nature Portfolio wishes to improve the reproducibility of the work that we publish. This form provides structure for consistency and transparency in reporting. For further information on Nature Portfolio policies, see our Editorial Policies and the Editorial Policy Checklist.

Statistics

For all statistical analyses, confirm that the following items are present in the figure legend, table legend, main text, or Methods section.

n/a	Confirmed	
☐	☒	The exact sample size (*n*) for each experimental group/condition, given as a discrete number and unit of measurement
☒	☐	A statement on whether measurements were taken from distinct samples or whether the same sample was measured repeatedly
☐	☒	The statistical test(s) used AND whether they are one- or two-sided<br>*Only common tests should be described solely by name; describe more complex techniques in the Methods section.*
☒	☐	A description of all covariates tested
☐	☒	A description of any assumptions or corrections, such as tests of normality and adjustment for multiple comparisons
☐	☒	A full description of the statistical parameters including central tendency (e.g. means) or other basic estimates (e.g. regression coefficient) AND variation (e.g. standard deviation) or associated estimates of uncertainty (e.g. confidence intervals)
☐	☒	For null hypothesis testing, the test statistic (e.g. *F*, *t*, *r*) with confidence intervals, effect sizes, degrees of freedom and *P* value noted<br>*Give P values as exact values whenever suitable.*
☒	☐	For Bayesian analysis, information on the choice of priors and Markov chain Monte Carlo settings
☒	☐	For hierarchical and complex designs, identification of the appropriate level for tests and full reporting of outcomes
☒	☐	Estimates of effect sizes (e.g. Cohen's *d*, Pearson's *r*), indicating how they were calculated

Our web collection on statistics for biologists contains articles on many of the points above.

Software and code

Policy information about availability of computer code

Data collection	No new data was collected and thus no software for data collection was used. The data was gathered from peer-reviewed journals using both WebPlotDigitizer and Engauge Digitizer.
Data analysis	We used MATLAB R2022b (MathWorks, Natick, MA) and IQM tools (IntiQuan GmbH, Basel, Switzerland), a continuation of SBTOOLBOX2, for modeling. IQM tools uses CVODES to numerically integrate the ODEs.<br>The parameter values were estimated using the enhanced scatter search (eSS) algorithm from the MEIGO toolbox.<br>The complete code for data analysis and modeling are available at https://github.com/willov/ameta static copy is available at Zenodo, DOI: 10.5281/zenodo.10104891.

For manuscripts utilizing custom algorithms or software that are central to the research but not yet described in published literature, software must be made available to editors and reviewers. We strongly encourage code deposition in a community repository (e.g. GitHub). See the Nature Portfolio guidelines for submitting code & software for further information.

Data

Policy information about availability of data

All manuscripts must include a data availability statement. This statement should provide the following information, where applicable:

- Accession codes, unique identifiers, or web links for publicly available datasets
- A description of any restrictions on data availability
- For clinical datasets or third party data, please ensure that the statement adheres to our policy

All data used for model estimation and validation can be accessed from the original publications. We provide all model related data files and parameter values in our public code repository, DOI: 10.5281/zenodo.10104891.

Research involving human participants, their data, or biological material

Policy information about studies with human participants or human data. See also policy information about sex, gender (identity/presentation), and sexual orientation and race, ethnicity and racism.

Reporting on sex and gender	This work only uses previously published data.
Reporting on race, ethnicity, or other socially relevant groupings	This work only uses previously published data.
Population characteristics	This work only uses previously published data.
Recruitment	This work only uses previously published data.
Ethics oversight	This work only uses previously published data.

Note that full information on the approval of the study protocol must also be provided in the manuscript.

Field-specific reporting

Please select the one below that is the best fit for your research. If you are not sure, read the appropriate sections before making your selection.

☒ Life sciences ☐ Behavioural & social sciences ☐ Ecological, evolutionary & environmental sciences

For a reference copy of the document with all sections, see nature.com/documents/nr-reporting-summary-flat.pdf

Life sciences study design

All studies must disclose on these points even when the disclosure is negative.

Sample size	This work only uses previously published data. The sample sizes and rationale are detailed in the original works.
Data exclusions	No data was excluded in this work.
Replication	This work only uses previously published data. We did not perform any reproducibility tests.
Randomization	This work only uses previously published data. We did not perform any additional randomization.
Blinding	This work only uses previously published data. For this work, additional blinding is not relevant.

Reporting for specific materials, systems and methods

We require information from authors about some types of materials, experimental systems and methods used in many studies. Here, indicate whether each material, system or method listed is relevant to your study. If you are not sure if a list item applies to your research, read the appropriate section before selecting a response.

Materials & experimental systems

n/a	Involved in the study
☒	☐ Antibodies
☒	☐ Eukaryotic cell lines
☒	☐ Palaeontology and archaeology
☒	☐ Animals and other organisms
☒	☐ Clinical data
☒	☐ Dual use research of concern
☒	☐ Plants

Methods

n/a	Involved in the study
☒	☐ ChIP-seq
☒	☐ Flow cytometry
☒	☐ MRI-based neuroimaging

Plants

Seed stocks: n/a

Novel plant genotypes: n/a

Authentication: n/a
